# On the Origin and Evolution of the Extant System of B Chromosomes in Oryzomyini Radiation (Rodentia, Sigmodontinae)

**DOI:** 10.1371/journal.pone.0136663

**Published:** 2015-08-25

**Authors:** Karen Ventura, Patricia Caroline Mary O’Brien, Camila do Nascimento Moreira, Yatiyo Yonenaga-Yassuda, Malcolm Andrew Ferguson-Smith

**Affiliations:** 1 Departamento de Genética e Biologia Evolutiva, Instituto de Biociências, Universidade de São Paulo, São Paulo, São Paulo, Brazil; 2 Cambridge Resource Centre for Comparative Genomics, Department of Veterinary Medicine, University of Cambridge, Cambridge, United Kingdom; 3 Instituto de Recursos Naturais - Universidade Federal de Itajubá, Itajubá, Minas Gerais, Brazil; Leibniz-Institute of Plant Genetics and Crop Plant Research (IPK), GERMANY

## Abstract

Heterogeneous supernumerary chromosomes (Bs) are recognized in the oryzomyines *Holochilus brasiliensis*, *Nectomys rattus*, *N*. *squamipes*, *Oligoryzomys flavescens* and *Sooretamys angouya*, representing about 10% of all known B-containing rodent species. They provide an outstanding model for understanding the origin, evolution and diversity of Bs in a phylogenetic context. Therefore, whole chromosome-specific probes were generated from flow-sorted *Holochilus brasiliensis* (HBR) autosomes 11 and 25+26 and chromosomes X, Y and Bs. Hybridizations were performed on male metaphases of 15 Oryzomyini species of which 3 are B-containing species. The results reveal that among the species sampled, 12 of them, belonging to a monophyletic Oryzomiyini subclade, are positive for an anonymous Oryzomyini shared heterochromatic region (*OSHR*) on both sex chromosomes. The *OSHR* is also present on Bs of *Holochilus brasiliensis*, *Nectomys rattus* and *N*. *squamipes* but not on Bs of *O*. *flavescens* and *S*. *angouya*. Two distinct additional *OSHR*/autosome associations are observed on *S*. *angouya*. The three species that are *OSHR* negative belong to an outgroup. Molecular dating suggests that the *OSHR* originated between 7.8 and 3 Mya on ancestral sex chromosomes. A tentative explanation for the *OSHR*-positive nature of B regions in three species could be that transposable elements (TEs) from this specific sex chromosome region may have invaded existing B chromosomes. The presence of the *OSHR* on entire Xp and Yp adjacent to interstitial telomeric sequences at pericentromeric positions, as observed in *Drymoreomys albimaculatus*, show a similar organization as on B chromosomes in *Nectomys squamipes*. The diversity of the Oryzomyini Bs in number, size, morphology and genetic content may be explained by the independent origin of B chromosomes in different subgroups of species, with Bs in *Holochilus brasiliensis*, *Nectomys squamipes* and *N*. *rattus* sharing the *OSHR* with sex chromosomes, and those in *Oligoryzomys flavescens* and *Sooretamys angouya* lacking *OSHR* in Bs. The species-specific pattern of Bs is probably a consequence of their independent evolutionary origin.

## Introduction

Supernumerary (B) chromosomes are found in about 15% of eukaryotic species, including animals, plants and fungi [1], and, in most cases, their genomic functions and molecular composition remain obscure [1,2]. The karyotypes of about 70 mammalian species have been characterized by the presence of B chromosomes, of which 50 species belong to the order Rodentia [3]. In Brazilian rodents, heterogeneous supernumeraries are known in the genome of nine species [4,5]. Among them, and representing about 10% of all known B-containing species of Rodentia, are *Holochilus brasiliensis*, *Nectomys squamipes*, *Nectomys rattus*, *Oligoryzomys flavescens* and *Sooretamys angouya*, belonging to the monophyletic Oryzomyini (Sigmodontinae), a group comprising 33 Neotropical genera, and about 140 species [6–8], which originated from a rapid adaptive radiation [9,10].

Oryzomyini have an exceptional range of diploid and fundamental numbers, with polymorphism of autosomes and sex chromosomes in some species [11–16]. Their B chromosomes show great diversity in terms of number, size, morphology of Bs, DNA replication, banding patterns and presence/absence of interstitial telomeric signals (ITS) [4,11].

Generally, two primary origins of B chromosomes have been considered [17,18]. The most widely accepted view is that they are derived from an intragenomic fragment of the autosomal (A) complement [19], as in the plants *Crepis capillaris* [20] and *Secale cereale* [19], in *Drosophila subsilvestris* [21] and *Abracris flavolineata* [22], in *Rattus rattus* [23] and in the Siberian roe deer *Capreolus pygargus* [24]. Although less well supported, B chromosomes could be derived from the sex chromosomes, as suggested in the case of the W chromosome in the New Zealand frog *Leiopelma hochstetteri* [25,26], and the Y chromosome in the collared lemming, *Dicrostonyx groenlandicus* [27]. An alternative view is that interspecific hybridization provides foreign DNA from a closely related species, as is presumed for the Amazon molly *Poecilia formosa* [28] and as demonstrated in two species of the wasp *Nasonia* [29]. The presence of Bs in some species is associated with amplification of certain genome regions and their initial accumulation within the population may occur as a result of meiotic drive (for review see [3]).

In mammals, supernumerary chromosomes have been investigated by FISH with telomeric or rDNA sequences, by chromosome microdissection, by BAC mapping and by DNA sequencing [23,24,30–35]. These modern approaches to investigating B chromosomes from animals and plants have improved our knowledge of their origin, composition, sequence accumulation, gene content and derived transcripts—for review see [36]. Although such different levels of DNA-sequence comparisons have been used to investigate the origin of Bs, in Oryzomyini they have been studied only for their similarity to autosomes and sex chromosomes in terms of morphology, size or meiotic behaviour.

## Materials and Methods

**Fig 1:**
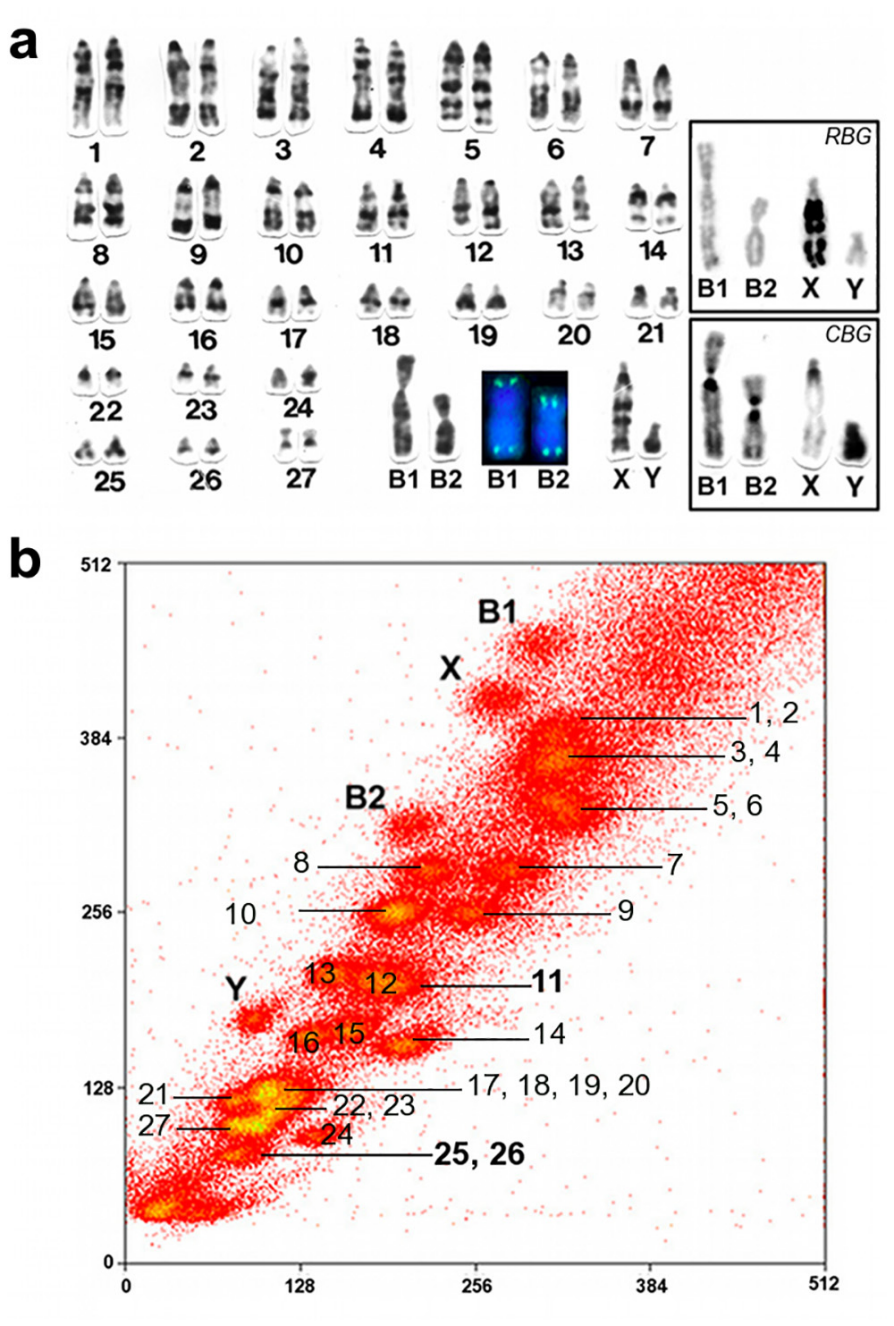
Karyotype and characterization of HBR probes. (a) GTG-banded karyotype of a male *Holochilus brasiliensis* (2n = 56+2Bs). Black square with B1 and B2 after telomeric FISH. Inset, supernumeraries B1 and B2, and sex chromosomes in RBG and CBG-banding pattern. (b) Flow karyotype of a male *Holochilus brasiliensis* (2n = 56+2Bs). The peaks containing the single HBR B1, B2, X, Y and 11 and the two chromosomes HBR 25, 26 are indicated in bold.

The whole chromosome-specific painting probes of *Holochilus brasiliensis* (HBR) HBR X, HBR Y, HBR supernumeraries (B1 and B2) and the two autosomal probes (HBR 11 and HBR 25, 26) were generated from flow-sorted chromosomes at the Cambridge Resource Centre for Comparative Genomics, Department of Veterinary Medicine, University of Cambridge, UK. The primary fibroblast cell lines came from one male *Holochilus brasiliensis* with 2n = 56+2Bs (Fig 1A and 1B). The chromosomes of HBR were arranged by size according to their position in the flow karyotype. On the hybridized metaphases, the HBR chromosomes were identified by 4′,6- diamidino-2-phenylindole (DAPI) differential staining. The chromosome-specific paints were made by degenerate oligonucleotide-primed polymerase chain reaction (DOP-PCR) on flow-sorted chromosomes [37, 38]. Briefly, the chromosomes were stained with Hoechst 33258 (2 μg/ml) and Chromomycin A3 (40 μg/ml) in the presence of magnesium sulfate (2.5 mmol/l) for 2 h. Sodium sulfite (25 mmol/l) and sodium citrate (10 mmol/l) were added 15 min prior to flow sorting. Chromosome sorting was performed using a dual-laser cell sorter (MoFlo; Beckman Coulter). Approximately 400 chromosomes were sorted from each peak in the flow karyotypes directly into PCR tubes containing 30 μl of distilled water. Each sample was amplified by DOP-PCR using the primer 6MW [37]. Primary PCR products were labeled using cyanine 3 (Cy3)-dUTP (GE Healthcare Lifesciences) or fluorescein isothiocyanate (FITC)-12-dUTP (Roche)–final concentration 0,1mM—by taking 1 μl of product to a second DOP-PCR using the same primer.

For cross-species hybridization, metaphases were obtained from cell cultures of male individuals belonging to 14 Oryzomyini species, as follows: *Cerradomys subflavus* (2n = 56), *Sooretamys angouya* (2n = 58+2 small heterochromatic metacentric Bs), *Pseudoryzomys simplex* (2n = 56), *Nectomys rattus* (NRA, 2n = 52+1 large submetacentric B), *Nectomys squamipes* (2n = 56+2 medium submetacentric Bs), *Drymoreomys albimaculatus* (DAL, 2n = 62), *Oligoryzomys nigripes* (2n = 62), *O*. *flavescens* (2n = 64+1 dot-like B), *O*. *fornesi* (2n = 62), *O*. *moojeni* (2n = 70), *Neacomys spinosus* (2n = 64), *Oecomys* sp. (2n = 80), *Hylaeamys megacephalus* (2n = 54) and *Euryoryzomys russatus* (2n = 80). The cultures came exclusively from cell lines deposited on the Cell bank "Banco de Células de Vertebrados do Laboratório de Citogenética" at Departamento de Genética e Biologia Evolutiva, Instituto de Biociências da Universidade de São Paulo. They were established between 1988 and 1992 and belong to an Institution recognized by CGEN (Brazilian Council of Genetic Heritage Management) as "Fiel Depositária de Amostras do Componente do Patrimônio Genético" or Trust Depositary of the Genetic Heritage Component Samples, and are accessible by permission (D.O.U.: 24/6/2003, Seção 1, P 119, http://www.ib.usp.br/temp.php).

Within species and cross-species hybridizations were performed according to Yang et al. [38]. First, denaturation of 1μl of the labeled HBR paints was performed at 75°C for 10 min in the presence of 10 μl of hybridization buffer (50% deionized formamide, 10% dextran sulfate, 20xSSC, 0.5 M phosphate buffer, pH 7.3). To reduce highly repetitive DNA signals pre-annealing was performed at 37°C for 30 min. The slides, prior to hybridization, were denatured in 70% formamide/2xSSC solution at 65°C for 2 min.

Same-species hybridizations were performed overnight at 37°C and cross-species hybridizations for 48h at 37°C. Post-hybridization washes included 2×5 min incubations in 50% formamide/2×SSC at 42°C followed by 2×5 min incubations in 2×SSC and immersion for 4 min in 4xSSC, 0.05% Triton X-100 “Sigma”). FISH with telomeric probes “Telomere PNA FISH Kit/FITC or Cy3 “Dako” was employed following the manufacturer’s protocol on HBR metaphases, and in conjunction with HBR B paints in DAL. The slides were counterstained with DAPI diluted with Vectashield and analysed with a Zeiss Axiophot fluorescence microscope equipped with software for image capture (Isis karyotyping system, MetaSystems). The results were discussed within the evolutionary context of the Oryzomyini radiation, considering the phylogenetic relationships and dating of the diversification of Sigmodontinae taxa as presented by Percequillo et al. [7], Avaria-Llautureo [39] and Parada et al. [40].

## Results

The hybridizations show the presence of homologous DNA segments on Bs in three of the five analysed Oryzomyini B-containing species following chromosome painting using whole HBR Bs, HBR X, HBR Y and HBR autosomes as probes.

The first results, using HBR B as probes, highlight partial homology among the Bs of *Holochilus brasiliensis*, *Nectomys squamipes* and *N*. *rattus*. No paint signal was detected either on the dot-like B of *Oligoryzomys flavescens* or on the Bs of *Sooretamys angouya*. These five species exhibited variable patterns of hybridization on specific regions of their X and Y chromosomes, and *Sooretamys angouya* also presents two autosome pairs partially painted by the HBR B probe. Given the specific HBR B painting patterns observed on segments of the sex chromosomes and on the autosomes of *S*. *angouya*, the genomes of the other 10 species (8 genera), without supernumeraries, were also included in the sample, giving a total of 15 species investigated by painting probes.

**Fig 2:**
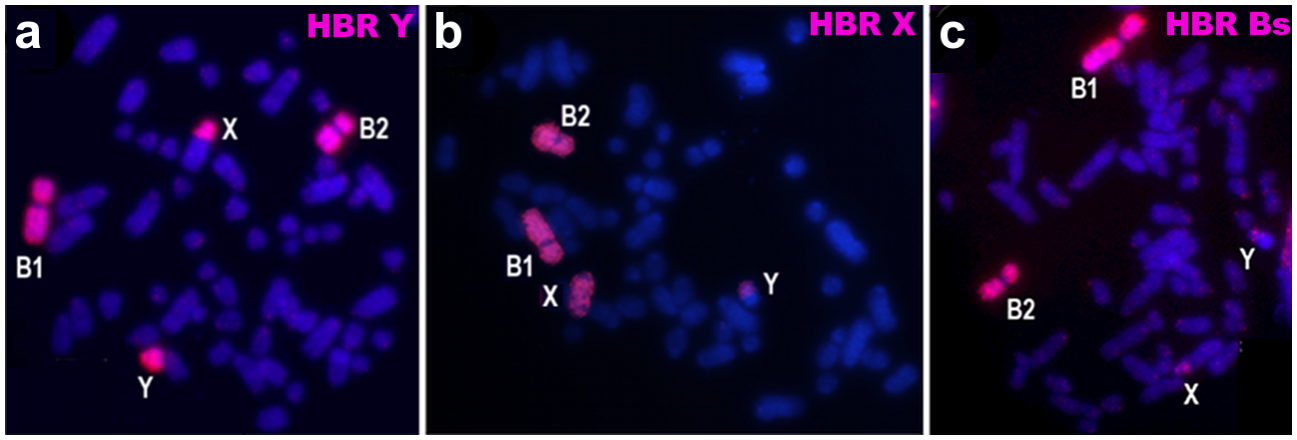
Same-species hybridization patterns on male *Holochilus brasiliensis* (2n = 56+2Bs) metaphases. (a) Whole HBR Y, HBR B1 and B2 and, region of HBR X painted by HBR Y probe; (b) Whole HBR X, HBR B1 and B2 and, region of HBR Y painted by HBR X probe. (c) Whole HBR B1 and B2, and regions of HBR Y and HBR X painted by HBR B1 or B2 probes. Painted chromosomes or regions are in pink.

The same-species hybridization using HBR Y and HBR X as probes exhibits signal on the whole HBR Y and pericentromeric region of HBR X (Fig 2a), and the whole HBR X and pericentromeric region of HBR Y (Fig 2b), respectively. Both probes hybridize to the whole HBR B1 and B2. HBR B probe hybridizes to regions of HBR X and HBR Y, in addition to both Bs (Fig 2c and Table 1).

**Table 1:** Name of the species, morphology of sex chromosomes, number and position of the painted *OSHR* segments on chromosomes X and Y and, numbers of Bs, number and position of the painted *OSHR* segments on Bs, after hybridization of HBR Y or HBR Bs chromosome-specific DNA as probes.

Species	Chro X	Number: Position	Chro Y	Number: Position	Bs	Number: Position	Figs
*C*. *subflavus*	A	1 seg: peri/prox Xq	A	1 seg: peri/prox Yq	-		3a
*S*. *angouya*	A	1 seg: peri Xq	A	1 seg: peri/prox Yq	2M	0 seg	3b and 4c
*H*. *brasiliensis*	St	1 seg: Xp/peri Xq	A	1 seg: peri/prox Yp	2SM	Entire Bs	2 and 3c
*P*. *simplex*	A	1 seg: peri Xq	A	2 seg: interst Yq + interst Yq	-		3d
*N*. *squamipes*	Sm	1 seg: Xp	A	1 seg: peri/prox	2SM	2 seg per B: Bp+distalBq	3e and 4b
*N*. *rattus*	Sm	1seg: Xp	_	-	1Sm	3seg: Bp+proxBq+distalBq	3f and 4a
*D*. *albimaculatus*	Sm	1seg: Xp	Sm	1seg: Yp	-		3g and 6a
*O*. *nigripes*	Sm	1 seg: Xp	Sm	1 seg: prox Yq	-		3h
*O*. *flavescens*	Sm	1 seg: Xp/peri Xq	A	1 seg: peri/prox	1dot	0 seg	3i
*O*. *fornesi*	A	1 seg: peri/prox Xq	A	2 seg: peri+dist Yq	-		3j
*O*. *moojeni*	Sm	1 seg: Xp/peri Xq	A	1 seg: peri/prox Yq	-		3k
*N*. *spinosus*	A	1 seg: peri Xp	Sm	2 seg: Yp+prox Yq	-		3l
*H*. *megacephalus*	*	-	*	-	-		Not shown
*E*. *russatus*	*	-	*	-	-		Not shown
*Oecomys* sp.	*	-	*	-	-		Not shown

A = acrocentric; Chro X = chromosome X; Chro Y = chromosome Y; dist = distal; interst = interstitial; peri = pericentromeric; prox = proximal; seg = segment; M = metacentric; Sm = submetacentric; St = subtelocentric; * = no hybridization occurred;—not applicable.

**Fig 3:**
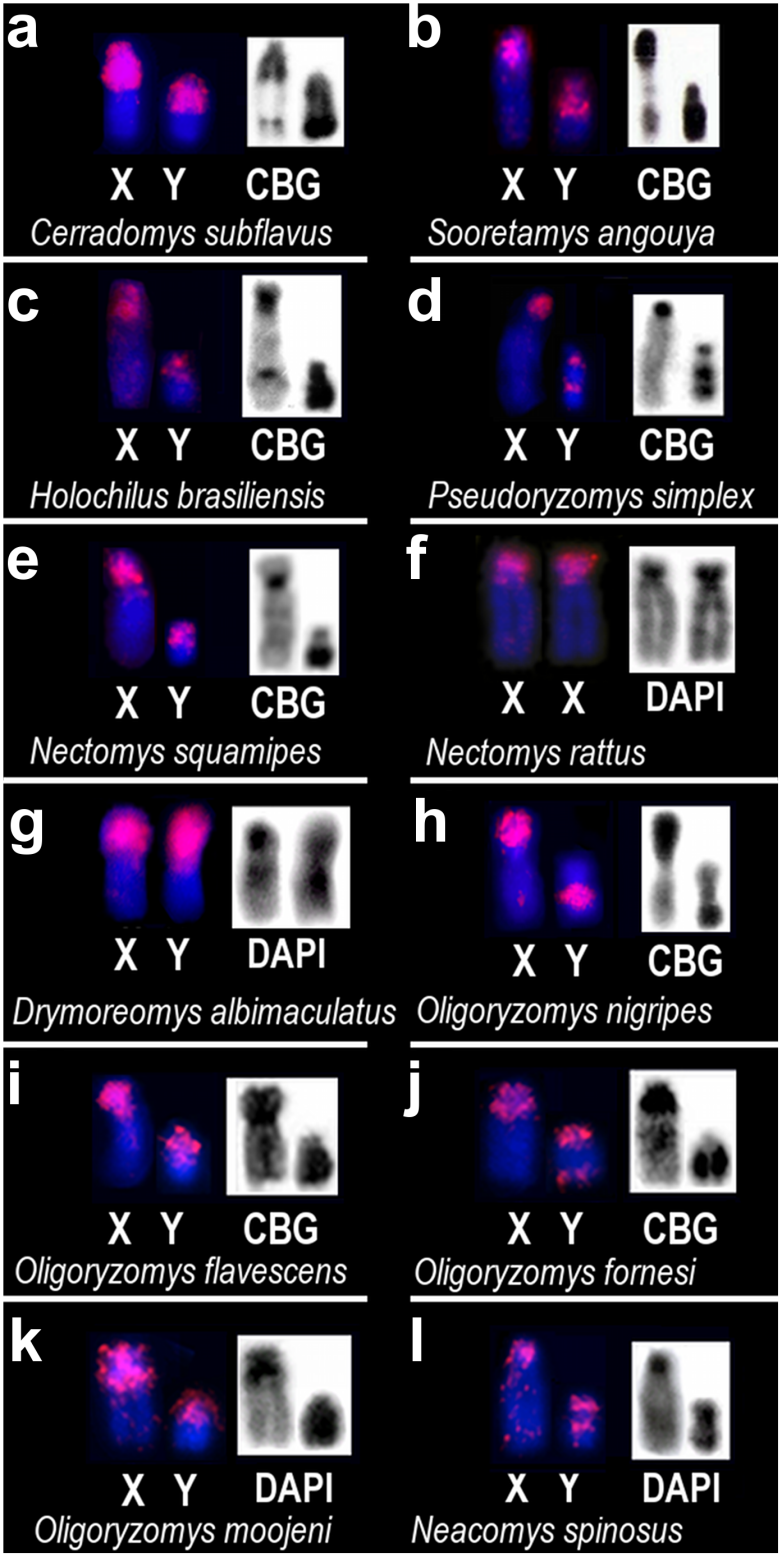
Cross-species painting patterns on sex chromosomes of 12 Oryzomyini species after *in situ* hybridization using HBR B1 or B2 or HBR Y (except in *Holochilus brasiliensis* for which the pattern of hybridization presented is exclusively obtained when HBR Bs are used as probes, see Fig 2c), as presented on Table 1: (a-l) On the left, X and Y painted regions indicating the *OSHR* in pink. On the right, the morphology of the sex chromosomes is shown by CBG-banding pattern or DAPI. Species names are indicated below the chromosomes. Species *Oecomys* sp., *Euryoryzomys russatus* and *Hylaeamys megacephalus* are outgroup *sensu* Percequilo et al. [7] and Avaria-Llautureo et al. [39], and their chromosomes did not present any painted region after HBR Y and HBR Bs paint probes hybridizations. See details of painted segments in Table 1.

**Fig 4:**
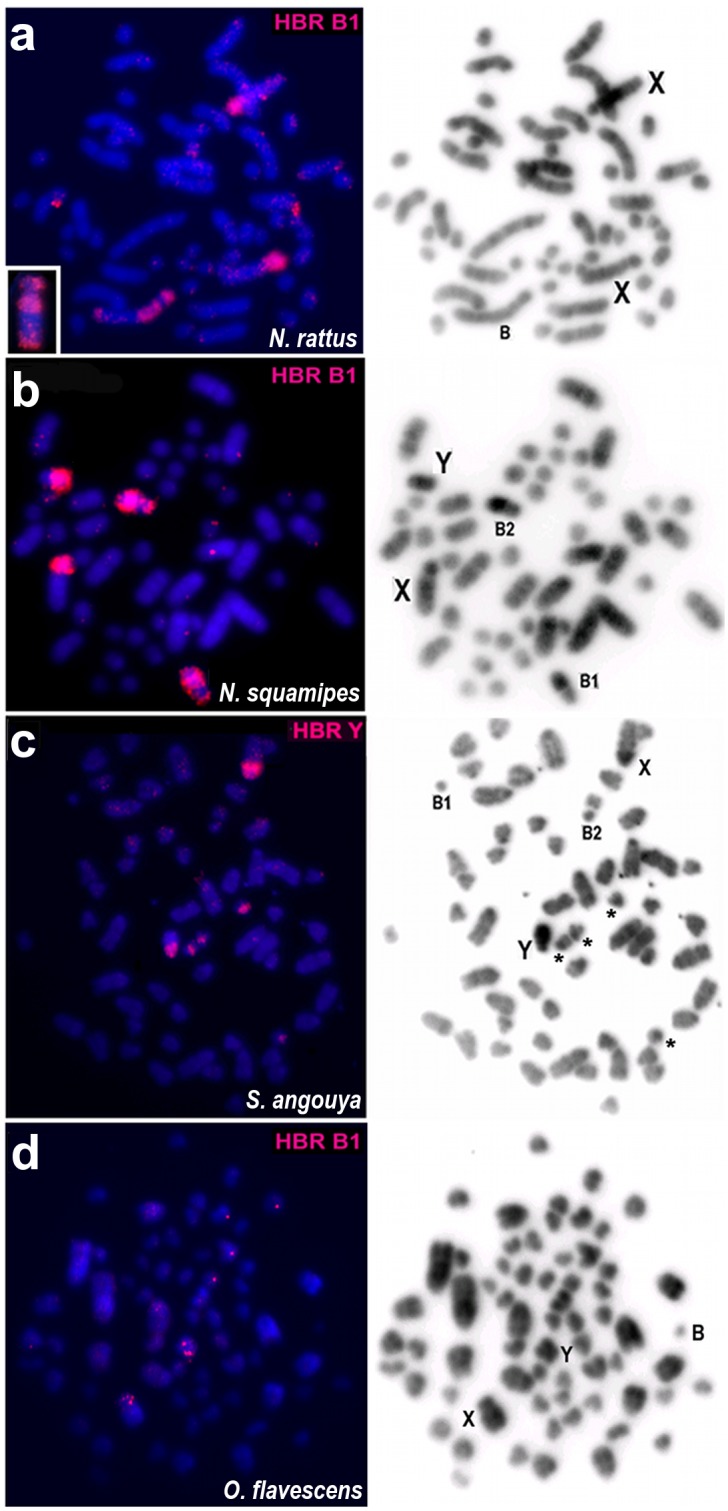
Cross- species hybridization on metaphases of B-containing species. (a) Hybridization of HBR B1 as probe on a female *N*. *rattus* (2n = 52+1B). In pink: positive signals on partial X (Xp and pericentromeric regions), and partial B (Bp, distal and proximal Bq, inset). (b) Hybridization on male of *N*. *squamipes* (2n = 56+2Bs) using HBR B1 as probe. Mostly B1 and B2 and partial X and Y are painted simultaneously, in pink. (c) Hybridization of HBR Y painting probe on a male of *Sooretamys angouya* (2n = 58+2Bs). The painted regions are in pink and localized at the pericentromeric regions of the X, Y and of four autosomes. Note that both small acrocentric supernumeraries (B1 and B2) are not painted. Asterisks indicate the autosomes. (d) Hybridization of HBR B1 as probe on a male of *O*. *flavescens*. Only partial X and Y exhibited positive signals. On the right side, the same metaphases are show after DAPI staining where X, Y and B indicated the respective chromosomes.

**Fig 5:**
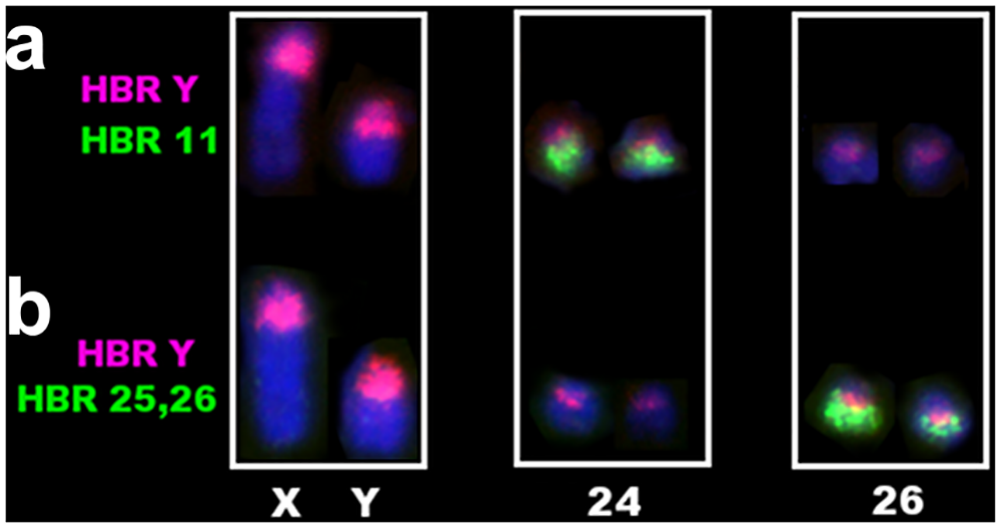
Patterns of hybridization on *Sooretamys angouya* X, Y and on the *OSHR*-containing autosomes 24 and 26. (a) Dual-colour hybridization on the *OSHR* positive X, Y, 24 and 26. (A) HBR B (red) and HBR 11 (green) signals on *S*. *angouya* chromosomes. Note the associated signal on chromosome 24 (b) Dual-colour hybridization using HBR B (red) and HBR 25, 26 (green) as probes. Observe the autosomal/*OSHR* (green/red) associations on chromosome 26. Chromosomes X, Y, 24 and 26 are indicated and the white frame insets homologous pairs from different metaphases.

HBR Y and HBR B paints gave congruent patterns of hybridization on both X and Y chromosomes in cross-species chromosome painting in all 12 species, except for *Oecomys* sp., *Hylaeamys megacephalus* and *Euryoryzomys russatus* in which no hybridization signals were observed (Fig 3 and Table 1). With respect to the B-containing species, congruent regions on Bs were also painted by both probes, except for *O*. *flavescens* and *S*. *angouya*, for which Bs did not show any hybridization signal, although two small acrocentric autosome pairs of *S*. *angouya* were partially painted by HBR Y and B paints (Figs 4 and 5). These autosomes are also partially homologous to the HBR 11 and HBR 25, 26 paints (Fig 5). Detailed results on the same and cross-species hybridizations using HBR Y or HBR B as probes are presented in Table 1. The HBR X-specific probe paints, in all analysed species, the entire X chromosome besides painting the same chromosome regions on Y and Bs as found when using HBR Bs as probes, (data not shown).

**Fig 6:**
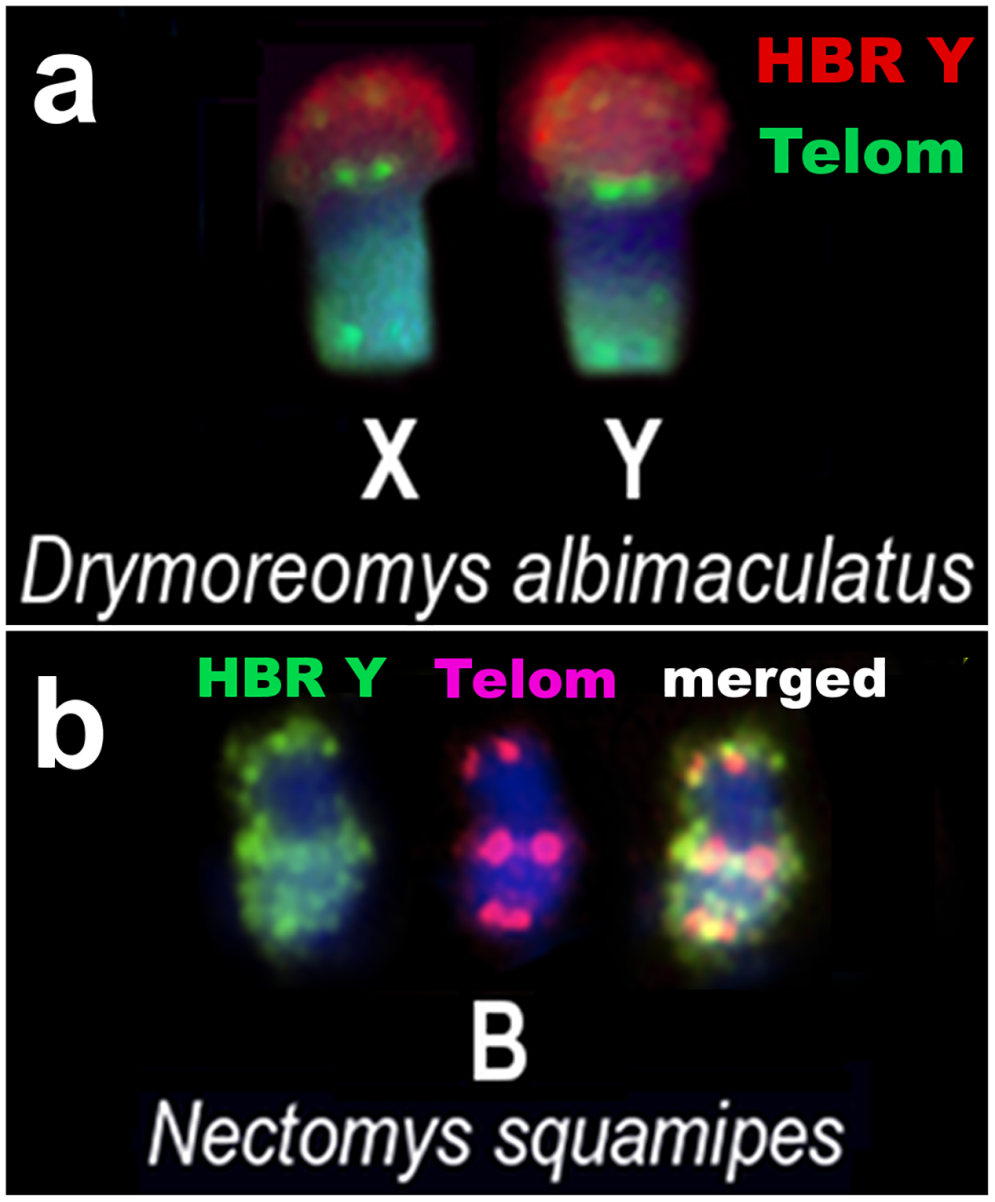
Characterization of *Drymoreomys albimaculatus* X and Y, and *Nectomys squamipes* B after hybridization using HBR Y and telomeric sequences as probes (a) Sex chromosomes of a male *Drymoreomys albimaculatus* after dual-colour FISH. HBR Y signals are red and telomeric sequences are green. (b) Medium metacentric B of *N*. *squamipes* after dual-colour FISH. HBR Y signals are green and telomeric sequences are red. Note the pericentromeric ITS linked to *OSHR* on the X and Y chromosomes of *D*. *albimaculatus* and on *N*. *squamipes* supernumerary.

Both sex chromosomes of *Drymoreomys albimaculatus* and the Bs of *Nectomys squamipes* show interstitial telomeric sites (ITS) adjacent to the HBR B and Y painting signals (Fig 6).

## Discussion

This study indicates the valuable contribution of chromosome painting to the recognition of an anonymous sequence shared by sex chromosomes, B chromosomes and autosomes in some species belonging to the Oryzomyini tribe. The shared region of homology, which is revealed after HBR B or Y hybridizations (hereafter termed the Oryzomyini shared heterochromatic region–*OSHR*) was not found in all Oryzomyini but only in 12 out of the 15 species analyzed. Remarkably, the 12 species showing the *OSHR* form a monophyletic clade arising during the Messinian period (about 6 million years ago) [7, 39, 40].

*OSHR* is therefore a synapomorphy for a node dated between 7 and 3 Mya according to Avaria-Llautureo [39] and between 7.8 and 4.8 Mya according Parada et al. [40], both corresponding to clades C and D reported by Percequillo et al. [7]. When present, the *OSHR* is localized in distinct regions of the X and Y chromosomes and also in the Bs of *H*. *brasiliensis*, *N*. *rattus* and *N*. *squamipes*. *S*. *angouya* showed *OSHR* in two acrocentric autosomes pairs in addition to regions of the X and Y chromosomes but not in the B chromosome.

### The origin of *OSHR*

We suggest a single and sex chromosome origin for *OSHR* since its presence in the X and Y is a synapomorphy in the species belonging to clade *OSHR* +.

The polymorphisms of *OSHR*, due to reshuffling or possible transposition principally on the Y chromosomes of *P*. *simplex*, *O*. *fornesi* and *N*. *spinosus* (Fig 3) argues in favour of these regions being TE donors once fragments emerge following translocation or transposition events.

The phylogenetic reconstructions presented by Parada et al. [40] show *D*. *albimaculatus* as sister to the genus *Eremoryzomys* in a basal position of the *OSHR* + clade. This species exhibits a particular organization of *OSHR* on the X and Y chromosomes when compared to the *OSHR* + species. *D*. *albimaculatus* sex chromosomes are similar in size, morphology and also in the amount and distribution of *OSHR* on Xp and Yp adjacent to terminal telomeric sequences and to the pericentromeric ITS (Fig 6a). A similar organization is observed in B chromosomes of *N*. *squamipes* and this suggests that *OSHR* may have led to the pericentromeric ITS (Fig 6b).

### The B chromosomes of Oryzomyini

Although the present data give support for a monophyletic origin of the *OSHR*, no evidence is found for a single origin of the extant B chromosomes in the Oryzomyini radiation since the four genera, including the species carrying B chromosomes (*Holochilus*, *Sooretamys*, *Nectomys* and *Oligoryzomys*), belong to different subclades. The additional fact that B chromosomes in *Sooretamys angouya* and *Oligoryzomys flavescens* lack *OSHR* is against a single origin of B chromosomes in this group.

The *OSHR* is shared by the Bs of *Holochilus* and the two *Nectomys* species, and the size of these particular segments is quite variable even between the two *Nectomys* species. Although a monophyletic origin for the Bs in *Nectomys* is possible, the homology found among them is only partial. The physical mapping of non-anonymous repetitive DNAs, such as satellite DNAs, gene families like rDNA (45S and 5S), histone genes, etc. could help to test this hypothesis since a monophyletic origin should imply a high number of shared DNA sequences between the two *OSHR*-painted Bs. However, their content differs between the two species within the same genera, in the absence and presence of ITS in *Nectomys squamipes* and *N*. *rattus* respectively.

The *OSHR* segments in the B chromosomes are variable in number, size and content, varying from whole Bs in *Holochilus brasiliensis* to parts of Bs in *Nectomys* (Table 1 and Figs 2 and 3). In *Nectomys squamipes* meiotic data confirm that Bs behave as univalents and are never found paired with autosomes or sex chromosomes [4]. As they may occur once, without recombination, Bs are thus free to proceed along independent evolutionary paths [23] giving rise to the diversity we find in Oryzomyini. The subsequent invasion of the B chromosomes by sequences or DNA families other than *OSHR* is also possible as proposed by Fantinatti et al. [41] for cichlid fishes.

A close relationship between sex and B chromosomes has been suggested among animals, and it is evident that they share some traits such as heterochromatin distribution, repetitive DNA accumulation and loss of gene activity [2]. Our results suggest a single common origin for *OSHR*, probably arising from 3 to 7 Mya, first exclusively as sex chromosome elements. A tentative explanation is that TEs in *OSHR* could be transposed to pre-existing Bs after their origin, or independently to A chromosomes. In this respect, although B chromosomes apparently share the *OSHR* region, actually the hybridization demonstrates only TEs contained in it. A comparative analysis of genomic sequences among sex, A and B chromosomes might help to elucidate this question. The heterogeneous system of Bs that we describe in *N*. *rattus*, *N*. *squamipes*, *Sooretamys angouya*, *O*. *flavescens* and *H*. *brasiliensis* seems to have evolved by independent drift through species-specific mechanisms of differentiation.
